# HSP90 Inhibitor PU-H71 in Combination with BH3-Mimetics in the Treatment of Acute Myeloid Leukemia

**DOI:** 10.3390/cimb45090443

**Published:** 2023-08-23

**Authors:** Katja Seipel, Scarlett Kohler, Ulrike Bacher, Thomas Pabst

**Affiliations:** 1 Department for Biomedical Research, University of Bern, 3008 Bern, Switzerland; scarlett.kohler@unibe.ch; 2 Department of Hematology, University Hospital Bern, 3010 Bern, Switzerland; veraulrike.bacher@insel.ch; 3 Department of Medical Oncology, University Hospital Bern, 3010 Bern, Switzerland; thomas.pabst@insel.ch

**Keywords:** acute myeloid leukemia (AML), B-cell lymphoma 2 (BCL2), cell surface glycoprotein CD34, stem cell factor receptor c-KIT (CD117), heat-shock protein 90 (HSP90), fms-like tyrosine kinase 3 (FLT3), myeloid cell leukemia 1 (MCL1), HSP90 inhibitor PU-H71, MCL1 inhibitor S63845, BCL2 inhibitor venetoclax

## Abstract

Targeting the molecular chaperone HSP90 and the anti-apoptotic proteins MCL1 and BCL2 may be a promising novel approach in the treatment of acute myeloid leukemia (AML). The HSP90 inhibitor PU-H71, MCL1 inhibitor S63845, and BCL2 inhibitor venetoclax were assessed as single agents and in combination for their ability to induce apoptosis and cell death in leukemic cells. AML cells represented all major morphologic and molecular subtypes including *FLT3-ITD* and *TP53* mutant AML cell lines and a variety of patient-derived AML cells. Results: PU-H71 and combination treatments with MCL1 inhibitor S63845 or BCL2 inhibitor venetoclax induced cell cycle arrest and apoptosis in susceptible AML cell lines and primary AML. The majority of the primary AML samples were responsive to PU-H71 in combination with BH3 mimetics. Elevated susceptibility to PU-H71 and S63845 was associated with *FLT3* mutated AML with CD34 < 20%. Elevated susceptibility to PU-H71 and venetoclax was associated with primary AML with CD117 > 80% and CD11b < 45%. The combination of HSP90 inhibitor PU-H71 and MCL1 inhibitor S63845 may be a candidate treatment for *FLT3*-mutated AML with moderate CD34 positivity while the combination of HSP90 inhibitor PU-H71 and BCL2 inhibitor venetoclax may be more effective in the treatment of primitive AML with high CD117 and low CD11b positivity.

## 1. Introduction

Heat-shock protein 90 (HSP90) is involved in the folding and maturation of a wide range of client proteins, including diverse kinases and transcription factors [1]. Over the last decade, HSP90 has gained attention due to its critical role in cancer. Treatment with HSP90 inhibitors may induce the destabilization of oncoproteins causally linked to the aberrant proliferation and survival of tumor cells. The associations between HSP90 and tumorigenesis indicate substantial therapeutic potential and many HSP90 inhibitors have been developed [2,3]. However, of all twenty compounds evaluated in clinical trials, due to HSP90 inhibitor toxicity and limited efficacy, none have been approved for clinical use as single agents [4]. Combining HSP90 inhibitors with other anticancer therapies might be a more advisable strategy to reduce toxicity and increase efficacy [5].

In acute myeloid leukemia (AML), a hyperactive signalosome drives addiction to oncogenic HSP90 species, thus rendering the cells vulnerable to HSP90-directed therapy [6]. PU-H71 (NSC 750424) is a purine-based inhibitor with notable specificity towards oncogenic HSP90 [7] with proven efficacy in a case report of an AML with high levels of epi-chaperone abundance [8]. The key oncogene in AML is the fms-like tyrosine kinase 3 (*FLT3*) growth factor receptor gene. FLT3-induced signaling pathways are highly active in AML cells, leading to elevated protein translation and cell proliferation as well as reduced apoptosis. In hematological malignancies including AML, tumor cells prevent apoptosis by the elevated expression of anti-apoptotic proteins of the B-cell lymphoma 2 (BCL2) family. Elevated levels of BCL2 protein in acute myeloid leukemia cells are associated with poor responses to chemotherapy [9]. *BCL2* mRNA is upregulated in 84% of AML patients at diagnosis and 95% at relapse [10]. The myeloid leukemia cell differentiation protein 1 (MCL1), another member of the BCL2 family proteins, is often upregulated in AML cells, particularly at relapse [11]. Hematological cells of various origins, including AML, exhibit specific dependencies on either BCL2, BCL-XL, or MCL1 for survival [12,13]. This dependency may be associated with the selective sequestration of the pro-apoptotic proteins BIM, BAX, and BAK by the specific anti-apoptotic BCL2 protein. BH3-mimetics displace pro-apoptotic BH3-containing proteins from their anti-apoptotic target. The BCL2 inhibitor venetoclax induced BAX-dependent apoptosis while the MCL1 inhibitor S63845 induced mainly BAK-dependent apoptosis. The MCL1 inhibitor S63845 specifically targets primary AML cells with elevated MCL1 protein levels [14]. S63845 has been proposed as a candidate treatment in AML in combination with the MEK inhibitor trametinib or the BMI1 inhibitor PTC596 in preclinical studies [15].

Here, we assessed the HSP90 inhibitor PU-H71, the MCL1 inhibitor S63845, and the BCL2 inhibitor venetoclax as single agents and in combination for their ability to induce apoptosis and cell death in leukemic cells in vitro. AML cells represented all major morphologic and molecular subtypes including the *FLT3-ITD* and *FLT3* wild type, *NPM1* mutant and wild type, and *TP53* mutant and wild type AML cell lines as well as a variety of patient-derived AML cells.

## 2. Materials and Methods

### 2.1. Patient Samples

Mononuclear cells of AML patients diagnosed and treated at the University Hospital, Bern, Switzerland, between 2015 and 2022 were included in this study. Informed consent was obtained according to the Declaration of Helsinki and the studies were approved by the decisions of the Ethics Committee of the Canton of Bern, Switzerland. Peripheral blood mononuclear cells (PBMCs) and bone marrow mononuclear cells (BMMCs) were collected at the time of diagnosis before the initiation of treatment. The AML cells were analyzed at the central hematology laboratory of the University Hospital Bern according to state-of-the-art techniques [16]. Conventional karyotype analysis based on conventional chromosome banding techniques, FISH, and array comparative genomic hybridization (aCGH) of at least 20 metaphases were performed in all samples. All samples were analyzed by NGS sequencing of the myeloid panel genes including *ASXL1, ASXL2, ATRX, BCOR, BCORL1, BRAF, CALR, CBL, CDKN2A, CEBPA, CREBBP, CSF3R, CSNK1A1, CTCF, CTNNA1, CUX1, DDX41, DNMT3A, EP300, ETV6, EZH2, FBXW7, FLT3, GATA1, GATA2, GNAS, HRAS, IDH1, IDH2, IKZF1, JAK2, KDM5A, KDM6A, KIT, KMT2D, KMT2C, KRAS, MLL, MPL, MYC, MYD88, NF1, NPM1, NRAS, PHF6, PPM1D, PTEN, PTPN11, RAD21, RB1, RUNX1, SETBP1, SF3B1, SH2B3, SMC1A, SMC3, SRSF2, STAG2, SUZ12, TET2, TP53, U2AF1, WT1, ZBTB7A*, and *ZRSR2*. Flow-cytometry immunophenotyping was conducted according to international consensus with CD markers in the initial evaluation of myeloid leukemia including CD7, CD11b, CD13, CD14, CD15, CD16, CD33, CD34, CD35, CD36, CD45, CD56, CD64, CD71, CD105, CD117, and HLA-DR.

### 2.2. AML Cell Lines

OCI-AML3 (AML-M4, *FLT3*wt, DNMT3A R882C, *NPM1*mut, *TP53*wt), MOLM-13 (AML-M5, t(9;11), *FLT3-ITD*, *TP53*wt), MOLM-16 (AML-M0, *FLT3*wt, *TP53*mut), ML-2 (AML-M4, t(6;11), *FLT3*wt, *TP53*wt), SKM-1 (AML-M5, *FLT3*wt, *TP53*mut), and PL-21 (AML-M3, *FLT3-ITD*, *TP53*mut) cells were supplied by the Leibniz Institute DSMZ, German Collection of Microorganisms and Cell Cultures. AML cells were grown in RPMI 1640 media (SIGMA-ALDRICH, St. Louis, MO, USA) supplemented with 20% fetal bovine serum (FBS, Biochrom GmbH, Berlin, Germany) in a standard cell culture incubator at 37 °C with 5% CO_2_.

### 2.3. Cytotoxicity Assays

The HSP90 inhibitor PU-H71 and the MCL1 inhibitor S63845 were purchased at MedChem-Express (Monmouth Junction, NJ, USA). A stock solution of Venetoclax was prepared by dissolving a tablet in DMSO (Venclexta^®^, Abbvie Inc., North Chicago, IL, USA). Cell viability was determined after 20 h of treatment using the MTT-based cell proliferation kit I (Roche Diagnostics GmbH, Mannheim, Germany). This time point was selected because the cellular responses were effectual for the calculation of combination indexes after 20 h of treatment with two compounds in leukemic cells. For AML cell lines, four independent assays (biological replicates) with four measurements (technical replicates) per dosage were performed. For hematological patient samples, two independent assays with three technical replicates per dosage were performed. The concentration of the drug resulting in 50% inhibition of cell viability (IC50) was calculated using three-parameter logistic curve fitting. For the calculation of combination indexes, three dosages of PU-H71 and two dosages of the other compounds were applied alone and in combination. Combination indexes were calculated on Compusyn software (version 1.0; ComboSyn, Inc., Paramus, NJ, USA). Data are depicted as XY graphs, column plots, or scatter plots with mean values and SD. Statistical analysis was performed on GraphPad Prism (version 9.5.1, GraphPad Software, San Diego, CA, USA) in grouped analysis and the significance was calculated by t-test for column graphs or Mann–Whitney test for scatter plots.

### 2.4. Measurement of mRNA Expression by qPCR

RNA was extracted from AML cells and quantified using qPCR. The RNA extraction kit was supplied by Macherey-Nagel, Düren, Germany. Reverse transcription was performed with MMLV-RT (Promega, Madison, WI, USA). Real-time PCR was performed on the Quant-studio 7 Flex PCR Instrument using FAST Start Universal probe master mix (Roche Diagnostics GmbH, Mannheim, Germany) and gene-specific probes (cat# 4331182, Thermo Fisher Scientific, Waltham, MA, USA): Hs00355782_m1 (*CDKN1A*), Hs01034249_m1 (*TP53*) and Hs02758991_g1 (*GAPDH*). Measurements for *CDKN1A* and *TP53* were normalized with *GAPDH* values (ddCt relative quantitation). Assays were performed in three or more independent experiments. Statistical analysis was performed on GraphPad Prism 9 software using unpaired t-tests. Data are depicted in column bar graphs with SD values.

### 2.5. Imaging Cytometry

Imaging cytometry was carried out on the NC-3000 cell analyzer (ChemoMetec, Allerod, Denmark) with reagents supplied by ChemoMetec. To determine the induction of apoptotis, cells were stained with AnnexinV-CF488A conjugate (Biotium, Fremont, CA, USA) in AnnexinV buffer and Hoechst 33,342 (10 μg/mL) for 15 min at 37 °C followed by several washes. Propidium iodide was added shortly before imaging. According to Annexin V and PI staining intensity, cells were classified as vital (Ann lo, PI lo), early apoptotic (Ann hi, PI lo), late apoptotic (Ann hi, PI hi), or necrotic (Ann lo, PI hi). For cell cycle analysis, cells were incubated in a lysis buffer with DAPI (10 μg/mL) for 5 min at 37 °C before imaging on the NC-3000 cell analyzer. According to DAPI staining, cell intensities were classified as subG1 (<2N), G0/G1 (2N), S phase (2–4N), or G2 phase (4N). Statistical analysis was performed using an unpaired t-test on GraphPad Prism 9 software. Data are depicted as column bar graphs with SD values.

### 2.6. Enzyme-Linked Immunosorbent Assay (ELISA)

Protein extraction was conducted according to the standard protocol. In short, cell pellets were lysed in RIPA buffer containing protease and kinase inhibitors. The protein concentration of the total lysate was calculated by a Bradford assay. Concentrations of specific proteins, namely FLT3, MCL1, and BCL2, were determined with double-antibody sandwich ELISA assays with a detection range of 0.312–20 ng/mL and intra-assay CV < 10% (SEA039Hu, SEC615Hu, SEA778Hu, Cloud-Clone Corp., Houston, TX, USA). Two independent assays with three technical replicates were performed per sample. Statistical analysis was performed using unpaired t-tests on GraphPad Prism 9 software. Data are depicted as column bar graphs with SD values.

## 3. Results

### 3.1. Variable Susceptibility of AML Cell Lines to HSP90 Inhibitor PU-H71

**Figure 1:**
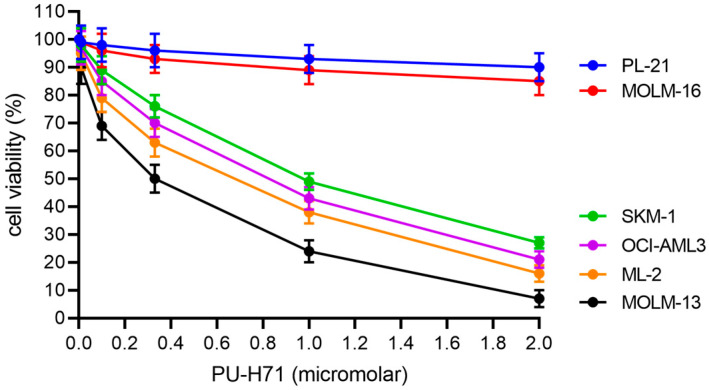
PU-H71 dose response in AML cell lines. AML cells were treated with the HSP90 inhibitor PU-H71 at the indicated dosages for 20 h. Cell viability data are average values of multiple repeat measurements per dosage.

To determine the sensitivity of AML cells to different targeted compounds, AML cells were subjected to in vitro cytotoxicity assays. Six AML cell lines were treated for 20 h in dose escalation experiments before cell viability assessment. Our panel of AML cell lines covered the majority of morphologic and molecular subtypes including *FLT3-ITD* and *FLT3* wild type, *NPM1* mutant and *NPM1* wild type as well as *TP53* wild type, mutant, and hemizygous cells (Table 1). The susceptibility to PU-H71 was elevated in the *FLT3-ITD* positive MOLM-13 cell line, with an approximate IC50 value of 0.3 micromolar; intermediate in OCI-AML3, ML-2, and *TP53* mutant SKM-1 cells, with IC50 values of 0.7 to 1.2 micromolar; and *TP53* mutant MOLM-16 and PL-21 cells were resistant with IC50 values above 10 micromolar (Figure 1, Table 2). IC50 values for S63845 and venetoclax were calculated, similar to a previous study [17]. With respect to S63845 and venetoclax, MOLM-13 cells were most susceptible with IC50 values of 0.02 and 0.1 micromolar, respectively, while MOLM-16 and PL-21 cells were resistant.

### 3.2. Combination Treatments in AML Cell Lines

**Figure 2:**
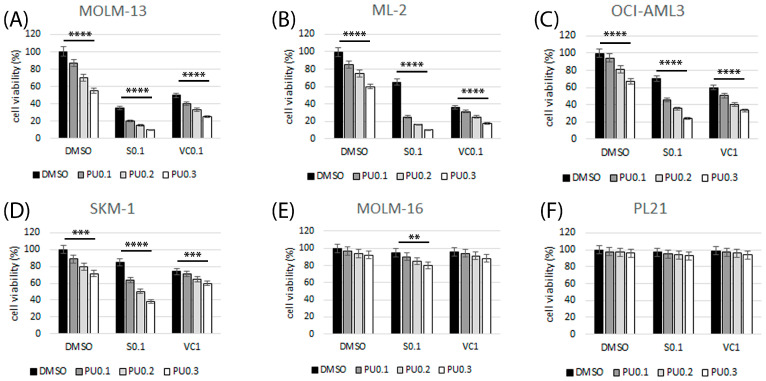
Susceptibility of AML cell lines to various treatment combinations. Cell viability was determined in AML cell lines MOLM-13 (**A**), ML-2 (**B**), OCI-AML3 (**C**), SKM-1 (**D**), MOLM-16 (**E**) and PL-21 (**F**) after 20 h of treatment with single compounds and in combination with 0.1–0.3 μM PU-H71 (PU) and 0.l μM S63845 (S0.1), 0.1 μM venetoclax (VC0.1), or 1 μM venetoclax (VC1). Significance of differences denoted for *p* < 0.01 (**); *p* < 0.001 (***); and *p* < 0.0001 (****).

In order to define the most effective treatment combinations, we focused on inhibitors expected to elicit synergistic effects in combination with the HSP90 inhibitor PU-H71 based on previous studies with MCL1 and BCL2 inhibitors [14,15,17,18]. Cell viability was determined in AML cell lines treated with increasing dosages of single compounds and in combination treatments using the HSP90 inhibitor PU-H71 and a variety of targeted therapies including the BCL2 inhibitor venetoclax and the MCL1 inhibitor S63845. Drug concentrations in the combination studies were chosen to correspond to minimally effective concentrations in single compound assays determined in initial titration. MOLM-13 and ML-2 cells were susceptible to 100 nM PU-H71, 100 nM S63845, or 100 nM venetoclax with enhanced effects on cell viability in the combination treatments (Figure 2A,B). OCI-AML3 and SKM-1 cells were susceptible to 100 nM PU-H71, 100 nM S63845, or 1 μM venetoclax with enhanced effects in combination treatments (Figure 2C,D). MOLM-16 and PL-21 cells were minimally affected by 100 nM PU-H71, 100 nM S638, and 1 μM venetoclax, with enhanced effects in the combination treatments (Figure 2E,F). Combination indexes were calculated according to the Chou–Talalay method [19]. Synergistic effects were present in the four susceptible cell lines with mild to moderate synergism of PU-H71 and venetoclax and moderate to strong synergism of PU-H71 and S63845 combination treatment (Table 3). Synergism appeared to be stronger in the combination of HSP90 inhibitor PU-H71 and MCL1 inhibitor S63845 compared to the combination with BCL2 inhibitor venetoclax.

### 3.3. Treatment Induced Cell Cycle Arrest, Protein Degradation, and Apoptosis

**Figure 3:**
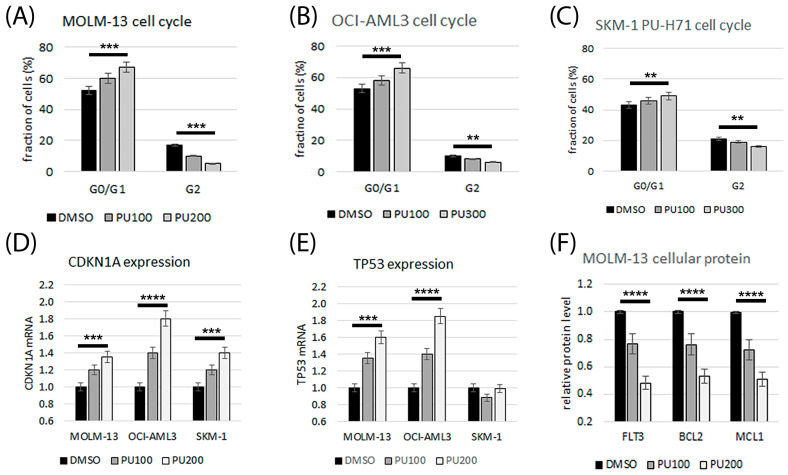
Induction of cell cycle arrest, gene expression, and protein degradation in AML cells treated with HSP90 inhibitor PU-H71 (PU). Cytometric analysis of AML cell lines MOLM13 (**A**), OCI-AML3 (**B**), and SKM-1 (**C**) after 20 h treatment with 100–300 nM PU-H71. According to DAPI staining, cell intensities were classified as the G0/G1 (2N) or G2 (4N) phase. Relative quantitation of *CDKN1A* (**D**) and *TP53* (**E**) gene expression. Relative quantitation of FLT3, BCL2, and MCL1 protein levels in MOLM-13 cells treated with 100–200 nM PU-H71 (**F**). Significance of differences denoted for *p* < 0.01 (**); *p* < 0.001 (***); and *p* < 0.0001 (****).

The effects of treatment with PU-H71 and S63845 or venetoclax alone and in combination on induction of apoptosis, cell cycle arrest, and cell death were determined in AML cell lines by cytometric analysis. Effects on the expression of cell cycle inhibitor CDKN1A and tumor suppressor TP53 were determined by qRT-PCR. PU-H71 treatment lead to G1 cell cycle arrest (Figure 3A–C) and induction of *CDKN1A* and *TP53* gene expression in the susceptible cell lines MOLM-13, OCI-AML3, and SKM-1 (Figure 3D,E). HSP90 inhibitor treatment may destabilize oncoproteins and cause degradation of cellular FLT3, BCL2, and MCL1 proteins [20,21]. Protein levels of FLT3, BCL2, and MCL1 were determined in AML cells treated with HSP90 inhibitor PU-H71, MCL1 inhibitor S63845, or BCL2 inhibitor venetoclax. FLT3, BCL2, and MCL1 protein levels were reduced in a dose-dependent manner in MOLM-13 cells treated with PU-H71 (Figure 3F). Changes in protein levels were also induced in AML cells treated with MCL1 inhibitor S63845 or BCL2 inhibitor venetoclax, with enhanced or reduced effects in the combination treatments (Figure S1).

**Figure 4:**
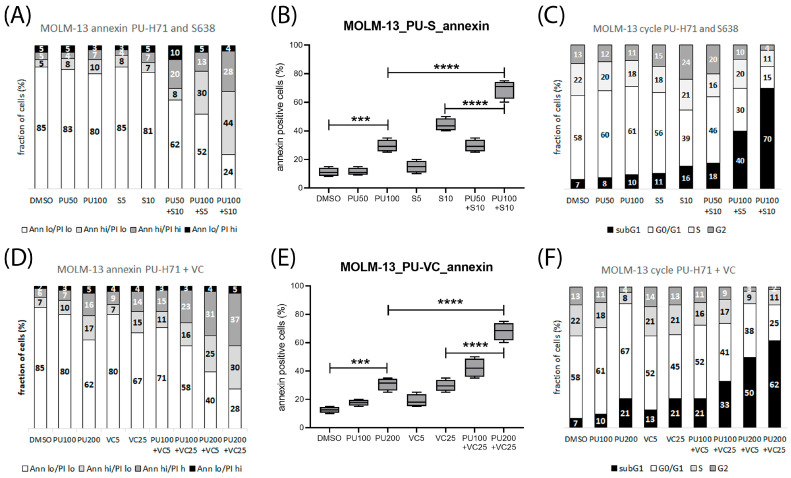
Induction of apoptosis and cell death in AML cells treated with PU-H71 in combination with S63845 or venetoclax. (**A**–**C**): Cytometric analysis of MOLM-13 cells after 20 h treatment with 50–100 nM PU-H71 and 5–10 nM S63845 (S) and stained with annexin-V and PI (**A**,**B**) or DAPI (**C**). (**D**–**F**): Cytometric analysis of MOLM-13 cells treated with 100–200 nM PU-H71 (PU) and 5–25 nM venetoclax (VC) and stained with annexin-V (**D**,**E**) or DAPI (**F**). According to Annexin V and PI staining intensity, cells were classified as vital (Ann lo, PI lo), early apoptotic (Ann hi, PI lo), late apoptotic (Ann hi, PI hi), or necrotic (Ann lo, PI hi). According to DAPI staining, cell intensities were classified as subG1 (<2N), G0/G1 (2N), S phase (2–4N) or G2 phase (4N). Significance of differences denoted for *p* < 0.001 (***); and *p* < 0.0001 (****).

There was an apparent S63845 treatment-induced reduction in FLT3 and BCL2 protein levels in both MOLM-13 and OCI-AML3 cells with an enhanced reduction in PU-H71 combination treatment. S63845 treatment led to a stabilization of MCL1 and a reduction in BCL2 protein in both cell lines. Venetoclax treatment led to reduced FLT3 protein levels in MOLM-13, but not in OCI-AML3 cells, and to reduced MCL1 protein levels in both cell lines. Apoptosis and cell death were induced in AML cells treated with PU-H71 and further enhanced in combination with the MCL1 inhibitor S63845 or the BCL2 inhibitor venetoclax (Figure 4 and Figure S2).

### 3.4. PU-H71 Combination Treatments in Leukemic Cells In Vitro

**Figure 5:**
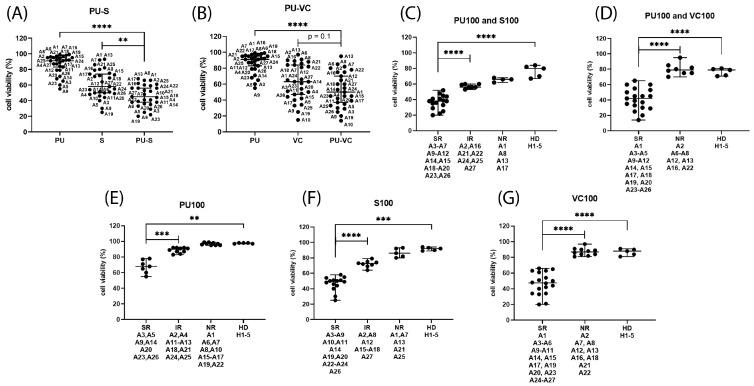
Hematological cells’ in vitro responses to PU-H71, S63845, and venetoclax. Cell viability was determined in mononuclear cells isolated from AML patients (A1-A27) and healthy donors (H1-H5) peripheral blood or bone marrow after 20 h treatment with 100 nM PU-H71 (PU), 100 nM S63845 (S), PU-H71 combined with S63845 (PU-S) (**A**), or PU-H71 combined with venetoclax (PU-VC) (**B**). The patient samples were sorted into response groups, namely substantial response (SR), intermediate response (IR), and minor (normal) response (NR) compared to healthy donors, treated with PU-H71 and S63845 (**C**), PU-H71 and venetoclax (**D**), 100 nM PU-H71 (**E**), 100 nM S63845 (**F**), and 100 nM venetoclax (**G**). The significance of differences in median values was calculated by Mann–Whitney test. Significance denoted for *p* < 0.01 (**); *p* < 0.001 (***); and *p* < 0.0001 (****).

After initial studies in AML cell lines, the treatment combinations of PU-H71 with S63845 or venetoclax were applied to patient-derived mononuclear cells isolated from peripheral blood (PB) or bone marrow (BM) and to mononuclear cells isolated from the peripheral blood of healthy donors. A total of 27 primary AML and 5 healthy donor cells (HD) were subjected to single compound and combination treatments (Table 4, Figure S3). Cytotoxic effects were mild to moderate in PU-H71 monotherapy, intermediate in S63845 or venetoclax monotherapy, and substantial in the combination treatment with PU-H71 and S63845 (Figure 5A). In contrast, the combination of PU-H71 and venetoclax did not significantly enhance cytotoxic effects compared to venetoclax monotherapy (Figure 5B).

The patient samples were sorted into response groups, namely substantial response (SR), intermediate response (IR), and minor (normal) response (NR); compared to healthy donor cells; and treated with 100 nM PU-H71 and 100 nM S63845 (Figure 5C), 100 nM PU-H71 and 100 nM venetoclax (Figure 5D), 100 nM PU-H71 (Figure 5E), 100 nM S63845 (Figure 5F), and 100 nM venetoclax (Figure 5G). The tested treatments induced a minor reduction in cell viabilities in mononuclear cells isolated from healthy donors and three primary AML samples. Substantial responses to PU-H71 monotherapy were observed in seven primary AML samples, all of which were *FLT3-ITD* positive AML. Intermediate and minor responses to PU-H71 monotherapy were detected in ten AML samples each. Fourteen primary AML samples had substantial responses to S53845 monotherapy, with nine *FLT3* mutated AML. Eight AML samples presented intermediate responses to S63845 monotherapy, with three *NRAS* and one *BRAF*-mutated AML. Seventeen primary AML samples had substantial responses to venetoclax monotherapy, with nine *FLT3* mutated AML. Ten AML and five healthy donor cells had minor responses to venetoclax.

According to the magnitude of in vitro response to combination treatment, the primary AML cells were grouped in four categories. C1: Elevated susceptibility to both combination treatments (17 of 27). C2: Substantial response to PU-H71 and S63845 but not to PU-H71 and venetoclax (6 of 27). C3: Substantial response to PU-H71 and venetoclax but not to PU-H71 and S63845 (2 of 27). C4: Minor response to both combination treatments (2 of 27). The two *TP53* mutated samples (A1 and A2) were in response categories C2 and C3. The four samples with different *PTPN11* mutations (A6, A8, A11, and A16) fell into four response categories, Y62D:C1, E69K:C2, F285I: C3, and A72V:C4. The six samples with *RAS/RAF* mutations (A7, A15, A16, A18, A21, and A27) fell into four response categories, one C1, two C2, two C3, and one C4. The eight samples with different *TET2* mutations fell into four response categories: one C1, three C2, one C3, and three C4. The only sample with an AML-ETO (A21) mutation was in response category C2.

### 3.5. Biomarkers of Responses to PU-H71 Combination Treatments in Leukemic Cells

**Figure 6:**
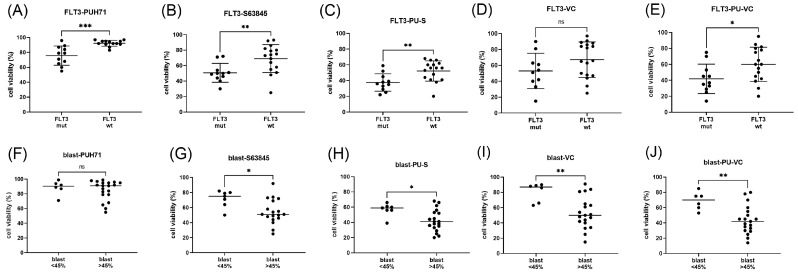
FLT3 status and blast cell percentage as biomarkers of responses to PU-H71 and BH3 mimetic combination treatments. Cell viability was determined in mononuclear cells isolated from AML patients’ peripheral blood or bone marrow after 20 h of treatment. Samples were grouped according to FLT3 genetic variation (**A**–**E**) or blast cell percentage (**F**,**G**). AML cells were treated in vitro with 100 nM PU-H71 (**A**,**F**), 100 nM S63845 (**B**,**G**), 100 nM venetoclax (**C**,**H**), PU-H71 combined with S63845 (**D**,**I**), or PU-H71 combined with venetoclax (**E**,**J**). The significance of differences in median values was calculated by a Mann–Whitney test. Significance of differences in median values was calculated by a Mann–Whitney test. Significance denoted for *p* < 0.05 (*); *p* < 0.01 (**); *p* < 0.001 (***); no significance denoted for *p* > 0.05 (ns).

**Figure 7:**
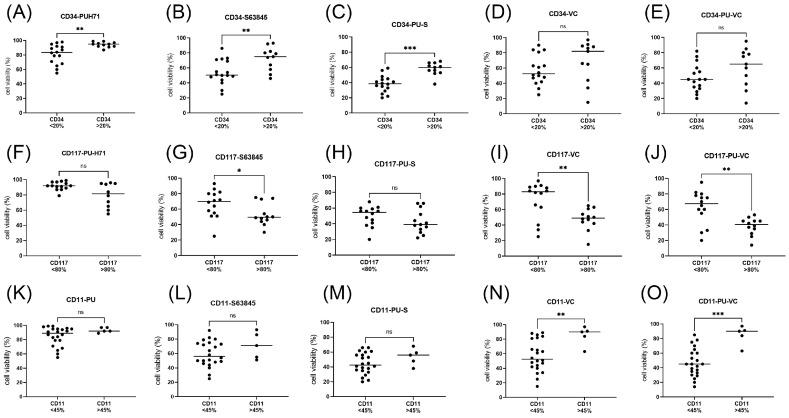
CD34, CD117, and CD11b biomarkers of responses to PU-H71 and BH3 mimetic combination treatments. Cell viability was determined in mononuclear cells isolated from AML patients’ peripheral blood or bone marrow after 20 h of treatment. Samples were grouped according to CD34 (**A**–**E**), CD117 (**F**–**J**), or CD11b (**K**–**O**) expression. Cells were treated in vitro with 100 nM PU-H71 (**A**,**F**,**K**), 100 nM S63845 (**B**,**G**,**L**), 100 nM venetoclax (**C**,**H**,**M**), PU-H71 combined with S63845 (**D**,**I**,**N**), or PU-H71 combined with venetoclax (**E**,**J**,**O**). Significance of differences in median values was calculated by Mann–Whitney test. Significance denoted for *p* < 0.05 (*); *p* < 0.01 (**); *p* < 0.001 (***); no significance denoted for *p* > 0.05 (ns).

Potential response markers were deduced from the correlation analysis of cell viabilities grouped according to diagnostic parameters including gene mutation status, peripheral blood and bone marrow blast cells percentage, and CD markers for the initial evaluation of myeloid leukemias including CD11b, CD34, and CD117 (c-KIT). *FLT3* status appeared to be the main biomarker of response to PU-H71 and S63845 treatment with elevated susceptibility of the *FLT3* mutated samples (Figure 6A–E). The blast cell percentage was positively associated to the response to BH3 mimetics S63845 and venetoclax with elevated susceptibility of AML samples with a blast cell count >45% (Figure 6F–J). The marker CD34 was negatively associated with the response to PU-H71 and S63845 treatment with elevated susceptibility of primary AML samples with CD34 < 20% (Figure 7A–E). The fifteen primary AML samples with low CD34 expression (CD34 < 20%) included nine *FLT3* mutated, two *RAS* mutated, and four *TET2* mutated AML. The eleven primary AML samples with elevated CD34 expression included five *TET2* mutated, three *RAS* mutated, three *PTPN11* mutated, and one *FLT3* mutated AML. CD117 marker was positively associated to venetoclax treatment response with elevated susceptibility of primary AML samples with CD117 > 80% (Figure 7F–J). The eleven primary AML samples with high CD117 expression (CD117 > 80%) included seven FLT3-mutated AML. The marker CD11b was negatively associated to responses to venetoclax treatment with elevated susceptibility of AML samples with CD11b < 45% (Figure 7K–O).

**Figure 8:**
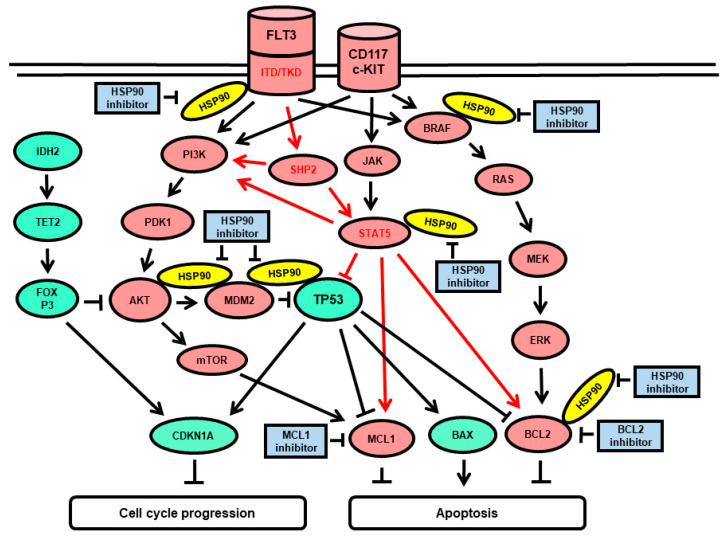
Schematic presentation of the FLT3 and c-KIT (CD117) induced signaling pathways and downstream effects. FLT3-ITD and FLT3-TKD are constitutively active growth factor receptors. C-KIT is an inducible growth factor receptor activated by stem cell factor (SCF) binding. Both tyrosine receptor kinases activate PI3K-AKT, RAS-MEK-ERK, and STAT5 leading to cell growth and proliferation via inhibition of the tumor suppressor TP53 and induction of the apoptosis regulators MCL1 and BCL2. HSP90 protein can bind and stabilize client proteins including AKT, BCL2, FLT3, JAK, MDM2, STAT5, SHP2, and BRAF. HSP90 proteins are indicated in yellow, oncogenic protein functions in red, tumor suppressor functions in green ovals, and targeted inhibitors in blue rectangles. Sharp arrows and blunt arrows indicate target induction and inhibition, respectively.

In summary, elevated susceptibility to PU-H71 and S63845 was present in FLT3 mutated AML with CD34 < 20% and to PU-H71 and venetoclax in primary AML with CD117 > 80% and CD11b < 45%. A possible scenario of intracellular signaling in AML cells affected by chemical inhibition of HSP90, BCL2, and MCL1 is presented in Figure 8.

## 4. Discussion

HSP90 inhibitors may destabilize oncoproteins associated with the cell cycle, angiogenesis, RAS-MAPK activity, histone modification, kinases, and growth factors. However, due to toxicity and limited efficacy, no HSP90 inhibitor has been approved for clinical use as a single agent. Combining HSP90 inhibitors with other anticancer therapies might be a more advisable strategy. Here, we assessed the HSP90 inhibitor PU-H71, the MCL1 inhibitor S63845, and the BCL2-inhibitor venetoclax as single agents and in combination for their ability to induce apoptosis and cell death in leukemic cells in vitro.

PU-H71 and combination treatments with MCL1 inhibitor S63845 or BCL2 inhibitor venetoclax induced cell cycle arrest and apoptosis in susceptible AML cell lines and primary AML samples. Drug induced changes in cellular protein levels were detected in AML cell lines treated with all three compounds. PU-H71 treatment led to reduced protein levels of FLT3, BCL2, and MCL1. Similar effects have been described where HSP90 inhibitor treatment destabilized oncoproteins and caused the degradation of cellular FLT3, BCL2, and MCL1 proteins [20,21]. S63845 treatment led to the stabilization of MCL1 protein and reduced BCL2 protein levels. A similar MCL1 protein stabilization was previously described as resulting from MCL1 protein half-life extension in the presence of S63845 [22]. The concurrent reduction in BCL2 protein may be a compensatory effect, as described in immune cells [23], leading to enhanced induction of apoptosis. Additionally, S63845 treatment led to reduced FLT3 protein levels in both MOLM-13 and OCI-AML3 cells. Other small molecules downregulating FLT3 protein expression have been reported [24]. Finally, Venetoclax treatment led to BCL2 protein stabilization and reduced MCL1 protein levels in both cell lines, again indicating a compensatory effect, leading to the enhanced induction of apoptosis.

The majority of the tested AML patient samples were susceptible to PU-H71 in combination with S63845, with few exceptions. Biomarkers of response were deduced from mutation profiles and target protein levels. In this study, FLT3 status appeared to be the main biomarker of response to HSP90-inhibitor PU-H71 and MCL1 inhibitor S63845 with elevated susceptibility of *FLT3*-mutated primary AML samples and the *FLT3-ITD*-positive cell line MOLM-13. FLT3-ITD is a constitutively active growth factor receptor signaling via PI3K-AKT, RAS-MEK-ERK, and STAT5, leading to cell growth and proliferation via p53 inhibition and MCL1 induction. Treatment with the HSP90 inhibitor PU-H71 may lead to degradation of client proteins including AKT, FLT3-ITD, STAT5, and BCL2 [1,20,21]. In addition, the cooperation of multiple BH3-only proteins (BID, BIK, and PUMA) and the suppression of the pro-survival BCL2 family member MCL1, via inhibition of STAT5A, may be involved [20], thus rendering the FLT3-mutated cells particularly susceptible to the HSP90 inhibitor. Blast cell percentage was positively associated with the response to BH3 mimetics S63845 and venetoclax. A significant association between the venetoclax response and elevated blast cell percentage was previously reported [25] and described with a boundary value of 60% peripheral blast percentage in the combination treatment with venetoclax and the PI3K inhibitor bimiralisib [17].

The marker CD34 was negatively associated with the response to PU-H71 and S63845 treatment with elevated susceptibility of primary AML samples with CD34 < 20%. CD34 is a cell surface selectin-binding glycoprotein with pro- or anti-adhesive function depending on its context and expressed glyco-form [26]. Leukemic stem cells are a subpopulation of leukemia cells characterized by the CD34+CD38- phenotype considered to be resistant to standard treatment [27,28]. High levels of CD34+CD38(dim)/CD123+ blasts indicate adverse prognosis in AML patients [29]. To address CD34+ adverse risk AML the combination of the BMI1 inhibitor PTC596 with the MCL1 inhibitor S63845 may be a more effective treatment option [15].

In the combination treatment with PU-H71 and BCL2 inhibitor venetoclax, two different markers were associated with responses: CD117 (c-KIT) and CD11b (MAC-1). CD117 was positively associated with a response with elevated susceptibility of primary AML samples with CD117 > 80%. CD-117 is a tyrosine kinase receptor expressed on the surface of hematopoietic stem cells (HSC), multipotent progenitors (MPP), and common myeloid progenitors (CMP) as well as on the majority of AML leukemic cells. Compared to CD34, which is expressed in leukemic cells of various origins, CD117 is a specific marker for leukemia of myeloid origin. Notably, both CD117 and FLT3-ITD can induce the same downstream signaling components including PI3K-AKT, RAS-MEK-ERK, and STAT5 (Figure 2). The marker CD11b was negatively associated with response to PU-H71 and venetoclax with elevated susceptibility of primary AML samples with CD11b < 45%. CD11b (MAC-1) is a neutrophil differentiation marker associated with AML therapy resistance [30,31]. Previous studies have shown that FAB-M5 patients lose expression of the primitive marker CD117 and up-regulate the expression of the monocytic marker CD11b [32] and monocytic subclones confer resistance to venetoclax-based therapy in AML patients [33].

Other genetic markers including *TP53* and *PTPN11* (SHP2) may also be associated with the treatment response. The tumor suppressor protein p53 and the cellular p53 inhibitor MDM2 are HSP90 clients. The conformations of both wild-type and mutant TP53 isoforms may be modified by HSP90 binding [34]. HSP90 inhibition may have opposing effects on wild-type and mutant p53 with downregulation of mutant p53 protein and upregulation of wild-type p53 [35]. Moreover, the inhibition of MDM2 by HSP90 may contribute to mutant p53 stabilization [36]. The SHP2 protein encoded by the *PTPN11* gene may also be an HSP90 client. HSP90 inhibition may have opposing effects on wild-type and mutant SHP2, with downregulation of wild-type SHP2 protein and upregulation of mutant SHP2 with varied effects depending on the specific amino acid change present in the particular SHP2 mutant protein. The vast majority of cases of de novo AML carry wild-type *TP53* and *PTPN11* alleles. Only 8% of primary AML cases carry mutated *TP53* or mutated *PTPN11* genes with single or multiple mutations with varied allele frequencies [37,38,39,40,41]. In order to address a potential association of treatment response to HSP90 inhibitors with *TP53* and *PTPN11* mutations in primary AML and to confirm the response markers CD34, CD117, and CD11b, preclinical studies in larger cohorts are required.

## 5. Conclusions

In this preclinical study, we assessed the HSP90 inhibitor PU-H71 in combination with the MCL1 inhibitor S63845 or the BCL2 inhibitor venetoclax in the treatment of acute myeloid leukemia and investigated the associated biomarkers of response. Our data suggest that both treatment combinations can effectively induce cell cycle arrest, apoptosis, and cell death in AML cells. Elevated susceptibility to PU-H71 and S63845 was present in *FLT3*-mutated AML with CD34 < 20% and to PU-H71 and venetoclax in primary AML with CD117 > 80% and CD11b < 45%. The combination of PU-H71 and BH3-mimetics may be effective in the treatment of AML with differential target specificity for MCL1 and BCL2 inhibitors. The combination of HSP90 inhibitor PU-H71 and MCL1 inhibitor S63845 may be a candidate treatment for *FLT3*-mutated AML with moderate CD34 positivity. The combination of HSP90 inhibitor PU-H71 and BCL2 inhibitor venetoclax may be a candidate treatment for primitive AML with high CD117 and low CD11b positivity.

## Figures and Tables

**Table 1:** Characteristics of leukemia cell lines.

ID	Disease	Status	*FLT3*	*TP53*	Gene Variants	Karyotype
ML-2	AML (M4)	de novo	wt	wt	KMT2A-AFDN KRAS A146T	t(6;11)
MOLM-13	AML (M5)	relapse	ITD	wt	KMT2A-MLLT3	t(9;11)
MOLM-16	AML (M0)	relapse	wt	V173M/ C238S	MLL V1368L	hypotetraploid
OCI-AML3	AML (M4)	de novo	wt	wt	DNMT3A R882C NRAS Q61L NPM1 L287fs	+1, +5, +8
PL-21	AML (M3)	de novo	ITD/ P336L	wt/ P36fs	KRAS A146V	hypertetraploid
SKM-1	AML (M5)	refractory	wt	R248Q/ R248Q	ASXL1 Y591* KRAS K117N	del(9q12)

**Table 2:** IC50 values (μM) in AML cell lines.

	Targeted Therapy		
Cell Line	PU-H71	Venetoclax	S63845
target	HSP90	BCL2	MCL1
ML-2	0.7	0.08	0.2
MOLM-13	0.3	0.1	0.02
OCI-AML3	0.9	1.2	0.2
SKM-1	1.2	2	0.4
MOLM-16	>10	>10	10
PL-21	>10	10	10

**Table 3:** Combination index values (CI) in AML cell lines.

Cell Line	PU-H71 Combination Treatment	
	S63845	Venetoclax
ML-2	0.1–0.3	0.5–0.7
MOLM-13	0.4–0.6	0.5–0.7
OCI-AML3	0.4–0.6	0.5–0.7
SKM-1	0.2–0.4	0.7–0.9
MOLM-16	0.3–0.5	1.1–1.3
PL-21	0.5–0.7	1.0–1.2

Combination indexes (CI) calculated according to the Chou–Talalay method [19]. Interpretation: CI = 0.1–0.3 strong synergy, CI = 0.3–0.7 moderate synergy, CI = 0.7–0.9 mild synergy; CI = 0.9–1.1 additive effects, CI > 1.1 antagonism.

**Table 4:** Clinical characteristics of hematological samples.

ID	Disease	Mutation Profile (Allelic Ratio, VAF%)	Karyotype	Source	CD34 %	CD117 %	CD11b %
A1	AML-M0	TP53 G245S (92%)	complex	BM	97	93	5
A2	AML-M4	FLT3-TKD (0.565), TP53 G245D (5%), NPM1 (19%), SRSF2 (47%), TET2 Q1357fs (42%), TET2 L1816fs (35%)	+8	PB	1	1	96
A3	AML-M5a	FLT3-ITD (0.5), NPM1 (49%), IDH2 (49%), DNMT3A (48%)	normal	BM	1	86	<1
A4	AML-M1/2	TET2 (47%), CEBPA (49%), GATA2 (14%)	normal	BM	6	86	1
A5	AML-M1/2	FLT3-ITD (1.1), NPM1 (43%)	normal	BM	4	86	8
A6	AML-M4	NPM1 (42%), PTPN11 E69K (40%)	nd	BM	6	16	86
A7	AML-M4	KRAS (64%), ASXL1 (42%), TET2 R1214W (32%)	normal	PB	1	1	100
A8	AML-M4/5	NPM1 (42%), DNMT3A (46%), PTPN11 A72V (31%), TET2 N275I (27%)	normal	PB	27	86	6
A9	AML-M1	FLT3-ITD (9.45), IDH2 (47%), NPM1 (48%)	normal	PB	20	95	40
A10	AML-NOS	FLT3 ITD (0.45), TET2 R1261H (47%), TET2 H1904R (48%), SRSF2 (54%)	normal	PB	97	99	3
A11	PV-AML	FLT3-TKD (0.16), PTPN11 Y62D (18%), IDH1 (42%), NPM1 (38%), SRSF2 (40%)	normal	PB	<1	98	10
A12	AML-M1	FLT3-ITD (0.56)	nd	PB	<1	80	5
A13	MDS-AML	TET2 Q278* (42%), TET2 M1701fs (35%), NPM1 (31%), ASXL (38%), SRSF2 (42%)	+8	PB	51	50	35
A14	AML-M4	FLT3-ITD (0.86), DNMT3A (45%), NPM1 (36%), SUZ12 (51%)	normal	PB	<1	84	7
A15	AML-M1	NRAS (45%), DNMT3A (46%), NPM1 (22%), RAD21 (44%)	+21	PB	<1	97	<1
A16	AML-M4	NRAS (32%), PTPN11 F285I (46%), DNMT3A S243fs (45%), DNMT3A M880V (48%)	der (7;14)	PB	87	20	12
A17	AML-M0	ASXL1 (48%), IDH2 (45%), RUNX1 (43%), SRSF2 (34%), STAG2 (9%)	+13	PB	98	94	1
A18	AML-M0	NPM1 (47%), TET2 H1382Q (48%), TET2 S1848* (45%), BRAF F595L (42%)	normal	PB	<1	4	4
A19	AML-M1	normal	normal	PB	16	20	<1
A20	AML-M1	FLT3 ITD (120.8), NPM1 (35%), WT1 R462P (47%)	normal	PB	19	90	10
A21	AML-M1	RUNX1-RUNX1T1 (AML1-ETO), NRAS (36%), RAD21 (34%)	t(8;21), -Y	PB	78	37	32
A22	AML-M2	SF3B1 (50%), TET2 S689fs*4 (50%), CBL (87%)	normal	PB	76	64	25
A23	AML-M1/2	FLT3 ITD (15.8), DNMT3A R882C (50%), NPM1 (39%), RUNX1 P263S (51%)	normal	PB	10	84	1
A24	AML-M4	DNMT3A V895M (46%), NPM1 (33%), IDH2 R140Q (46%)	normal	PB	<1	30	57
A25	AML-M5	normal	+8	PB	55	44	45
A26	AML-M1	FLT3 ITD (0.3), ZBTB7A (25%)	normal	PB	1	74	<1
A27	MDS-AML	TET2 N338fs (50%), TET2 S405fs (49%), EZH2 (96%), NRAS (46%), SMC1A (49%), ASXL1 (42%)	normal	PB	71	77	1
H1-H5		normal	normal	PB	<1	nd	nd

Abbreviations: variant allele frequency (VAF), bone marrow (BM), peripheral blood (PB), polycytemia vera (PV), myelodysplatic syndrome (MDS), not determined (nd).

## Data Availability

Data are available on request due to restrictions, privacy, and ethics.

## Supplementary Materials

The following supporting information can be downloaded at: https://www.mdpi.com/article/10.3390/cimb45090443/s1. Figure S1 Treatment induced changes in the levels of cellular FLT3, BCL2 and MCL1 proteins. Figure S2. Induction of apoptosis and cell death in AML cells treated with PU-H71 in combination with S63845 or venetoclax. Figure S3. Hematological cells in vitro response to PU-H71, S63845 and venetoclax.
