# Supplementary material for: HSP90 Inhibitor PU-H71 in Combination with BH3-Mimetics in the Treatment of Acute Myeloid Leukemia
Source: Curr Issues Mol Biol. 2023 Aug 23;45(9):7011–26. doi: 10.3390/cimb45090443 (PMC10529370; doi:10.3390/cimb45090443)
Supplement: Supplementary file 1 [file cimb-45-00443-s001.zip › cimb-2552398-supplementary-done.pdf]

*current issues in molecular biology*

MDPI

*Supplementary materials*

# HSP90 Inhibitor PU-H71 in Combination with BH3-Mimetics in the Treatment of Acute Myeloid Leukemia

**Katja Seipel, Scarlett Kohler, Ulrike Bacher and Thomas Pabst**

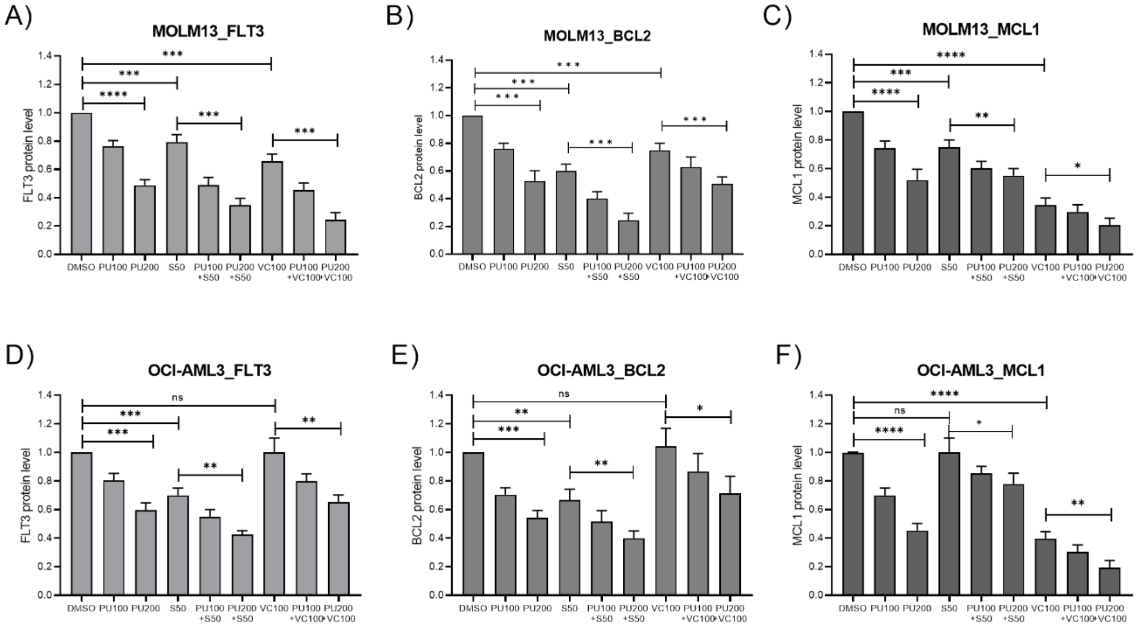


**Figure S1.** Treatment induced changes in the levels of cellular FLT3, BCL2 and MCL1 proteins. Relative quantitation (ELISA) of protein levels in AML cell lines MOLM-13 (A,B,C) and OCI-AML3 (D,E,F) after 20 h treatment with 100-200nM PU-H71 (PU), 50nM S63845 (S) or 100nM venetoclax (VC). Protein levels are shown for untreated cells (DMSO), single compound and combination treated cells. Significance denoted for $p < 0.05$ (*); $p < 0.01$ (**); $p < 0.001$ (***); no significance denoted for $p > 0.05$ (ns).

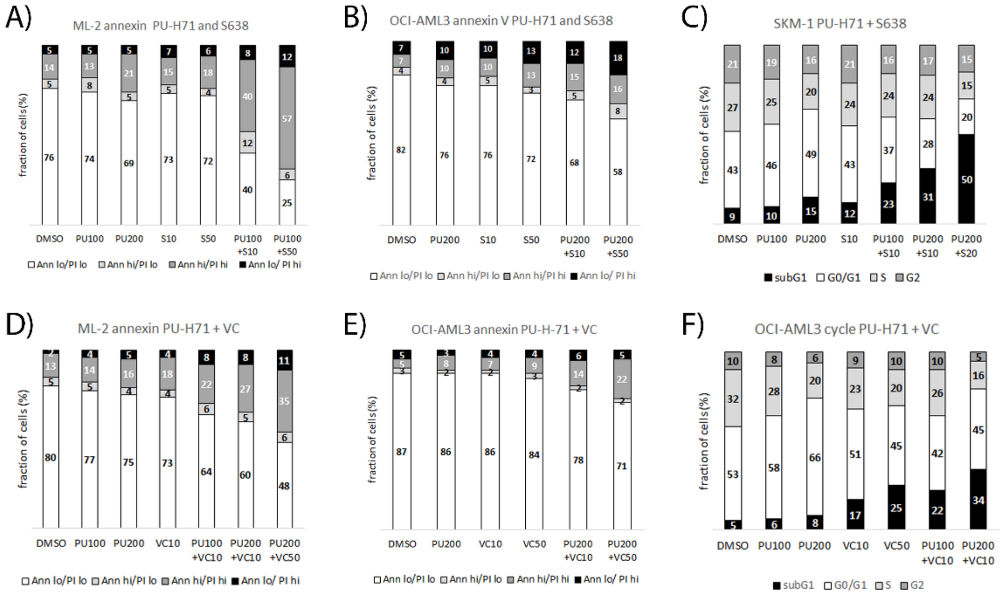


**Figure S2.** Induction of apoptosis and cell death in AML cells treated with PU-H71 in combination with S63845 or venetoclax. A-C: Cytometric analysis of AML cells after 20h treatment with 100-200nM PU-H71 and 10-50nM S63845 (S) and stained with annexin-V and PI (A,B) or DAPI (C). D-F: Cytometric analysis of AML cells after 20h treatment with 100-200nM PU-H71 (PU) and 10-50nM venetoclax (VC) and stained with annexin-V (D,E) or DAPI (F). AML cell lines ML-2 (A,D), OCI-AML3 (B,E,F) and SKM-1 (C). According to Annexin V and PI staining intensity, cells were classified as vital (Ann lo, PI lo), early apoptotic (Ann hi, PI lo), late apoptotic (Ann hi, PI hi) or necrotic (Ann lo, PI hi). According to DAPI staining intensity cells were classified as subG1 (<2N), G0/G1 (2N), S phase(2-4N) or G2 phase (4N).

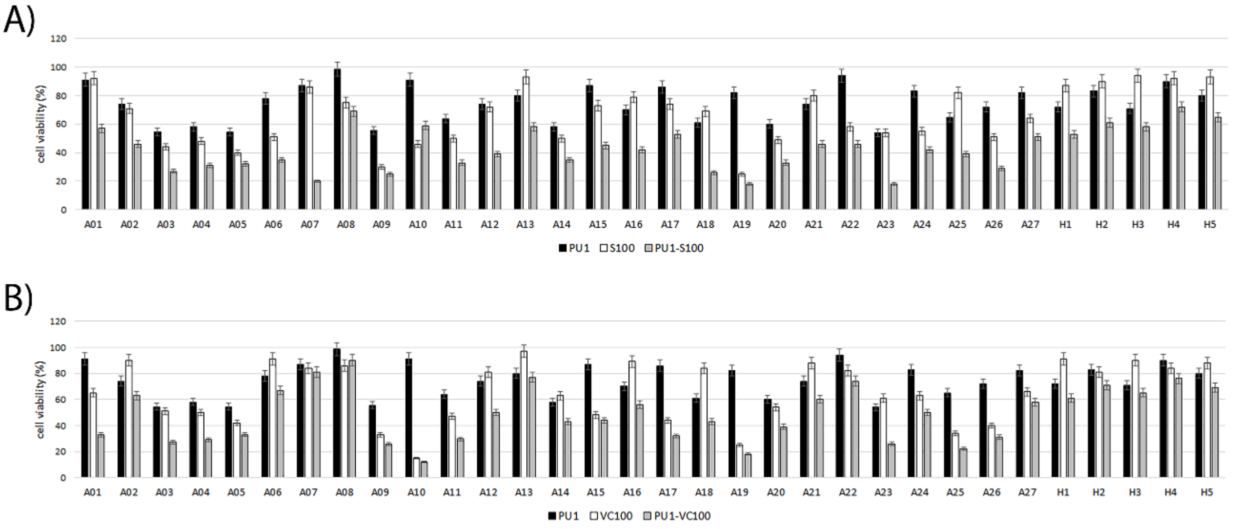


**Figure S3.** Hematological cells in vitro response to PU-H71, S63845 and venetoclax. Cell viability was determined in mononuclear cells isolated from AML patients (A1-A27) or healthy donors (H1-H5) peripheral blood or bone marrow after 20 h treatment. A) Cell viability in AML cells treated with 100nM PU-H71 (PU), 100nM S63845 (S), PU-H71 combined with S63845 (PU-S). B) Cell viability in AML cells treated with 100nM PU-H71 (PU), 100nM venetoclax (VC) , or PU-H71 combined with venetoclax (PU-VC).
